# Severe hypercalcaemia and lymphoma in an HTLV-1 positive Jamaican woman: a case report

**DOI:** 10.1186/1752-1947-1-56

**Published:** 2007-07-25

**Authors:** Veronica Lyell, Elham Khatamzas, Theresa Allain

**Affiliations:** 1 Department of Care of the Elderly, Southmead Hospital, Bristol, UK

## Abstract

Human T cell lymphotrophic virus type-1 infection is endemic in the Afro-Caribbean community in Britain, with carriage rates of about 3%. Although there is a long latency, carriers have a 1–5% chance of developing adult T cell leukaemia/lymphoma, a condition frequently complicated by marked and refractory hypercalcaemia, and with a poor prognosis. We present the case of an elderly Jamaican woman with severe hypercalcaemia and a raised PTHrP who was found to have lymphoma and was positive for HTLV-1.

## Case presentation

An 81-year-old Jamaican woman, who had lived in the UK for many years, presented with a four week history of progressive malaise, anorexia, weakness, nausea, vomiting, drowsiness and confusion. Her only past history was of longstanding falls and dizziness. She had been taking Calcium/vitamin D tablets and prochlorperazine.

**Figure 1:**
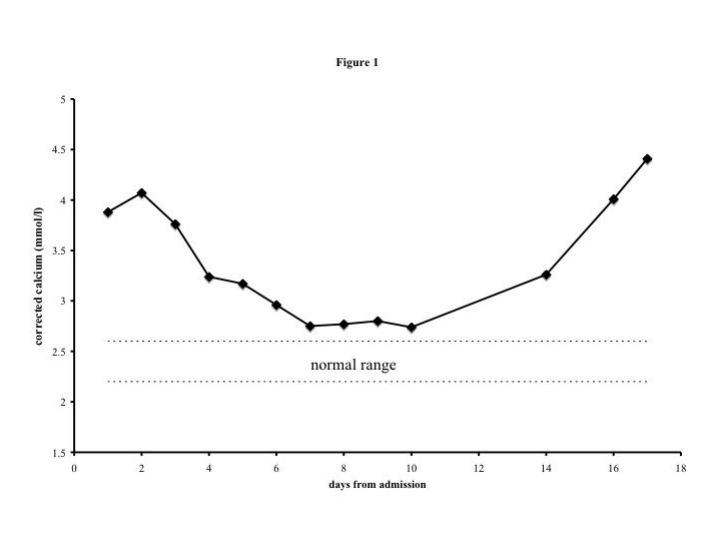
Serum calcium concentration over the course of the admission.

On admission she was drowsy, with a slightly distended and tender abdomen. Otherwise, physical examination was normal. Abdominal ultrasound showed no organomegaly or lymphadenopathy. Investigations revealed extreme hypercalcaemia with a corrected calcium of 4.07 mmol/l (figure 1, reference range 2.2–2.6 mmol/l). Phosphate was normal and alkaline phosphatase (liver isoenzymes) elevated at 323 IU/L (20–110), with an albumin of 27 g/l (35–50). Her renal function, thyroid function, full blood count and chest radiograph were normal. Parathyroid hormone (PTH) was suppressed at 0.9 pmol/l (1.48–7.63); serum angiotensin converting enzyme levels and serum and urine protein electrophoresis were normal. Her lactate dehydrogenase (LDH) was elevated at 808 IU/l (285–540). Bone scintigraphy showed some generalised increased bone uptake, suggesting metabolic bone disease, but no focal abnormality suggestive of metastases. The parathyroid hormone-related peptide (PTHrP) was elevated at 2.5 units (normal < 1.8).

Emergency management of hypercalcaemia including hydration, loop diuretics and iv pamidronate led to initial improvement in her serum calcium level and conscious level.

**Figure 2:**
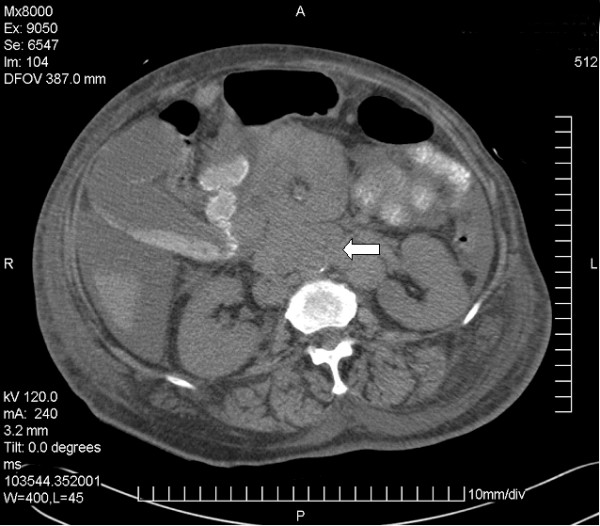
**CT of upper abdomen**. Extensive lymphadenopathy is noted in the para-aortic area (arrow) and surrounding the superior mesenteric artery. Body wall oedema, ascites and gall bladder sludge reflect the patient's debilitated condition.

However, over the following week, she developed extensive palpable lymphadenopathy. A CT of the chest, abdomen and pelvis revealed massive lymphadenopathy in the supraclavicular, axillary, mediastinal, retrocrural, mesenteric and para-aortal regions highly suggestive of disseminated lymphoma (figure 2). Tru-cut biopsy of a cervical lymph node was technically unsuccessful and subsequently a fine needle aspiration sample showed features consistent with non-Hodgkin's lymphoma (NHL). Excision biopsy was cancelled due to clinical deterioration in the patient.

Her calcium level, which had initially responded to therapy, rose rapidly again to 4.41 mmol/l. In consultation with the haematologists she was given high dose steroids, but she continued to deteriorate, with a high fever. A decision was reached, with her family, to provide palliative care only and she died shortly afterwards.

In view of the co-existence of lymphoma and hypercalcaemia, with elevated PTHrP, in this woman of Jamaican origin, Human T cell lymphotrophic virus type-1 (HTLV-1) serology was sought, and was positive, giving a presumptive diagnosis of HTLV-1-induced acute adult T cell leukaemia/lymphoma (ATLL).

## Discussion

ATLL is an aggressive malignancy that is aetiologically linked with the infection caused by HTLV-1[1]. HTLV-1 infection is endemic in Japan, the Caribbean and parts of Africa [2]. Transmission is from lymphocyte to lymphocyte in breast milk, semen or blood transfusion[3]. Prevalence rises with age and is approximately 3% in British Jamaicans. Hence there are potentially 22, 000 infected people in the UK, predominantly older Afro-Carribeans[4]. 1–5% of carriers develop ATLL, with a latency of 10–30 years[3,4].

The virus belongs to the oncovirus subfamily of retroviruses and can immortalise human lymphocytes, specifically CD4 positive T lymphocytes in ATLL[1]. Acute ATLL is invariably fatal, with a mean survival of 6 months. Prognosis is worse where there is poor performance status, age over 40, an elevated serum calcium, high level of LDH, and a higher tumour bulk.

In about 70% of cases, severe and refractory hypercalcaemia complicates acute ATLL and is one of the main causes of early death (by contrast, fewer than 4% of Hodgkin's Disease and NHL cases are associated with hypercalcaemia)[2,5]. PTHrP, which was elevated in our patient, plays a key role in the humoral hypercalcaemia of malignancy. The peptide binds to the PTH receptor and increases both calcium levels (through bony resorption and calcium reabsorption in the kidney) and the production of pro-inflammotory cytokines, stimulating IL-6 from osteoblasts and IL-8 and TNF-a from non-bony tissue such as normal immune cells[6]. High levels of inflammatory cytokines also potentiate the hypercalcaemic effect of PTHrP, and stimulate further PTHrP production. We were able to demonstrate raised PTHrP levels in our patient with ATLL and hypercalcaemia. ATLL patients also often express receptor activator of NF-kB ligand (RANKL), which cooperates with macrophage colony-stimulating-factor to stimulate haematopoietic precursors into osteoclasts. This effect, and the high levels of PTHrP, give rise to widespread bony resorption [2,7] and our patient's bone scan is consistent with this.

PTHrP levels are not affected by bisphosphonate therapy[8] and the management of the refractory hypercalcaemia of ATLL is limited. However, there are case reports of the successful use of somatostatin analogues in reducing PTHrP and calcium levels in other tumours[9,10]. Recently, a monoclonal antibody against PTHrP has been shown to block PTHrP function and reduce calcium levels in mouse models of hypercalcaemia[11].

Abnormal liver function, as in our patient's case, is frequent in ATLL and results from malignant liver infiltration, though in NHL liver function is rarely affected.

Those affected by ATLL also display a degree of immunodeficiency, with impairments in T-cell function allowing for opportunistic protozoal and fungal infections. HTLV-1 carriers have high rates of *Strongyloides stercoralis *infection[12]. In ATLL this gut pathogen is often associated with hyperinfection and fatal gram-negative bacteraemia[3], although in our patient there was no evidence of this.

Current chemotherapeutic regimens fail to alter the survival rates in ATLL, despite often inducing an initial remission. There are however reports of response to antiretroviral therapies, and of some successes in allogenic haematopoietic stem cell transplantation. Monoclonal antibodies against ATLL cells are also being developed[2,3].

## Conclusion

This case describes the presentation and clinical course of lymphoma in a woman from a population where HTLV-1 infection is endemic. The specific abnormalities associated with our patient's lymphoma, particularly the hypercalcaemia, raised PTHrP and abnormal liver function are all typical of ATLL associated with HTLV-1 and we were able to confirm posthumously that she was sero-positive for HTLV-1 infection.

Earlier identification of the aetiology is unlikely to have changed the outcome in this case, but the combination of lymphoma with hypercalcaemia in patients from endemic areas should alert physicians to the possibility of this diagnosis.

Key learning points are:

1) Hypercalcaemia is a common medical problem with a large number of potential causes, but is rarely associated with lymphoma.

2) Adult T cell leukaemia/lymphoma (ATLL) is frequently complicated by refractory hypercalcaemia largely due to raised PTHrP.

3) ATLL is rare, but much commoner in populations where HTLV-1 is endemic (in Britain, chiefly the Afro-Caribbean community).

## Competing interests

The author(s) declare that they have no competing interests.

## Authors' contributions

VL, EK and TJA were all involved in managing the case and in preparing the report manuscript. All authors read and approved the final manuscript.
